# The potential of miR-153 as aggressive prostate cancer biomarker

**DOI:** 10.1016/j.ncrna.2022.10.002

**Published:** 2022-10-13

**Authors:** Irina Gilyazova, Elizaveta Ivanova, Mikhail Sinelnikov, Valentin Pavlov, Elza Khusnutdinova, Ilgiz Gareev, Aferin Beilerli, Ludmila Mikhaleva, Yanchao Liang

**Affiliations:** a Institute of Biochemistry and Genetics, Ufa Federal Research Center of the Russian Academy of Sciences, 450054, Ufa, Russia; b Bashkir State Medical University, 450008, Ufa, Russia; c Sechenov First Moscow State Medical University (Sechenov University), 119435, Moscow, Russia; d Avtsyn Research Institute of Human Morphology of FSBI “Petrovsky National Research Centre of Surgery", 117418, Moscow, Russia; e Department of Obstetrics and Gynecology, Tyumen State Medical University, 54 Odesskaya Street, 625023, Tyumen, Russia; f The First Affiliated Hospital of Harbin Medical University, Harbin, 150001, China

**Keywords:** Gene expression, Aggressive prostate cancer, miR-153, Target gene mutations, Oncogenesis, Biomarker

## Abstract

**Introduction:**

Prostate cancer (PC) is one of the most frequently diagnosed cancers in males. MiR-153, as a member of the microRNA (miRNA) family, plays an important role in PC. This study aims to explore the expression and possible molecular mechanisms of the miR-153 action.

**Methods:**

Formalin-fixed paraffin-embedded (FFPE) tissues were collected from prostatectomy specimens of 29 metastatic and 32 initial stage PC patients. Expression levels of miR-153 were measured using real-time reverse transcription polymerase chain reaction (qRT-PCR). 2−ΔΔCT method was used for quantitative gene expression assessment. The candidate target genes for miR-153 were predicted by TargetScan. Mutations in target genes of miR-153 were identified using exome sequencing. Protein-protein interaction (PPI) networks, Kyoto Encyclopedia of Genes and Genomes (KEGG) analyses were performed to investigate the potential molecular mechanisms of miR-153 in PC.

**Results:**

MiR-153 was significantly up-regulated in PC tissues compared to non-cancerous tissues. The analysis of correlation between the expression level of miR-153 and clinicopathological factors revealed a statistically significant correlation with the stage of the tumor process according to tumor, node, metastasis (TNM) staging system (p = 0.0256). ROC curve analysis was used to evaluate the predictive ability of miR-153 for metastasis development and it revealed miR-153 as a potential prognostic marker (AUC = 0.85; 95%CI 0.75–0.95; sensitivity = 0.72, specificity = 0.86)). According to logistic regression model the high expression of miR-153 increased the risk of metastasis development (odds ratios = 3.14, 95% CI 1.62–8.49; p-value = 0.006). Whole exome sequencing revealed nonsynonymous somatic mutations in collagen type IV alpha 1 (COL4A1), collagen type IV alpha 3 (COL4A3), forkhead box protein O1 (FOXO1), 2-hydroxyacyl-CoA lyase 1 (HACL1), hypoxia-inducible factor 1-alpha (HIF-1A), and nidogen 2 (NID2) genes. Moreover, KEGG analysis revealed that the extracellular matrix–receptor (ECM-receptor) interaction pathway is mainly involved in PC.

**Conclusion:**

MiR-153 is up-regulated in PC tissues and may play an important role in aggressive PC by targeting potential target genes.

## 1 Introduction

Prostate cancer (PC) is a commonly diagnosed condition, with an estimated 1414.3 thousand new cases of PC and 375.3 thousand deaths from the disease in 2020 [1]. In one third of PC patients the tumor progresses (perineural and stromal invasion) after initial regression in response to androgen deprivation therapy [2]. Despite current advances in surgical, chemotherapeutic and radiological methods of treatment, the five-year survival rate in castration-resistant patients is approximately 31.0% [3]. Most PC-related deaths occur due to the inability of existing treatments to prevent tumor spread [4].

Rectal examination and prostate specific antigen (PSA) levels are used to diagnose PC. However, the medical and scientific communities have questioned routine PSA testing. PSA has high sensitivity but very low specificity for PC. It can be elevated in the presence of benign prostate disease, infection, inflammation or benign hyperplasia [5]. Routine PSA testing leads to a high percentage of false-positive results. Moreover, PSA levels correlate poorly with the stage of the disease, leading to misdiagnoses and overtreatment of indolent forms of PC [6]. Considering all the above, there is a need for new molecular genetic markers capable of both detecting the disease at the earliest stage and predicting its course.

MicroRNAs (miRNAs) are considered to be one of the most promising markers for various diseases including PC. The discovery of miRNAs provided a conceptual breakthrough in cancer research. MiRNAs are non-coding RNAs (ncRNAs) (19–22 nucleotides) which are involved in post-transcriptional regulation of gene expression. MiRNAs are increasingly associated with the initiation, development and progression of malignancies. Aberrant miRNAs expression has been identified in a variety of malignant tumors, with recent evidence suggesting that miRNAs function as tumor suppressor genes and/or oncogenes [7]. Recent studies have various miRNAs expression profiles in PC tissues [[8], [9], [10], [11]]. MiR-153 was found to be downregulated in various cancers, such as breast cancer (BC), gastric cancer (GC) and oral cancer (OC) [15]. However, there are few investigations devoted to miR-153 and its role PC [[12], [13], [14], [15]].

In this study, we evaluate the expression levels of miR-153 in PC tissue specimens and adjacent normal tissue specimens, the association of miR-153 with clinical characteristics and mutations in target genes of miR-153 and identified the pathways involved in PC progression. Our study was aimed to evaluate and understand the expression level of miR-153 in malignant tumor and normal prostate tissue in patients with metastatic PC and initial stages of the disease and possible molecular mechanism of the miR-153.

## 2 Material and methods

### 2.1 Patients and samples collection

In this study, 61 PC patients (average age 60, range 41–80 years) who underwent surgical treatment between 2008 and 2020 in Bashkir State Medical University hospital were included. Ethical approval for this study was obtained from Institute of Biochemistry and Genetics Bioethics Committee. All samples investigated in this study were obtained with written informed consents of the participants. We collected formalin-fixed paraffin-embedded (FFPE) tissues from prostatectomy material including 29 metastatic and 32 localized (stages I, II) PC patients. The samples were classified using the tumor, node, metastasis (TNM) staging system from clinical stages I-IV.

### 2.2 Samples evaluation

After tissue section were obtained, Hematoxylin and Eosin (H&E) staining was performed and the slides were examined by two independent experienced pathologists. DNA and RNA were isolated from tumor regions and normal prostate tissue from each patient. The healthy region of the prostate without tumor cells in the selected FFPE block, was taken as a source of normal RNA and DNA. Total RNA and DNA extraction was performed using Quick-DNA/RNA™ FFPE Kits (Zymo Research) following the manufacturer's protocol. The process consisted of simple tissue deparaffinization in deparaffinization solution, proteinase K treatment followed by RNA and DNA isolation in spin columns.

### 2.3 Real-time reverse transcription polymerase chain reaction (qRT-PCR)

The PCR amplification for the quantification of the miR-153 and U6 RNA was performed using a TaqMan MicroRNA Reverse Transcription Kit (Applied Biosystems; Life Technologies Corp) and TaqMan Human MicroRNA Assay Kit (Applied Biosystems; Life Technologies Corp). Expression levels were measured using CFX96™ PCR detection system (BioRad). All reactions were performed three times for each sample. The 2−ΔΔCT method was used for quantitative gene expression assessment. The 2−ΔΔCT method is based on the assumption that the cycle threshold difference (ΔCt) between target gene and reference gene is proportional to relative target gene expression. The relative expression of miR-153 was shown as fold difference relative to U6 RNA.

### 2.4 Whole exome sequencing

Patients with metastatic adenocarcinoma were chosen for exome sequencing to identify mutations in target genes of miR-153. Target genes were selected using TargetScan. DNA fragmentation, library preparation, and exome capture were conducted according to the manufacturer's recommendations. Selection of specific DNA fragments was conducted using the SureSelect system followed by concurrent sequencing of the obtained libraries using Illumina HiSeq 2000. All the reads were aligned with the reference genome using Burrows-Wheeler Alignment (BWA) software. We used the human genome sequence (Genome Reference Consortium Human Build 37 (GRCh37-hg19)) as a reference. Identification of the variants was conducted using the Genome Analysis Tool Kit (GATK). The identified variants were annotated by ANNOVAR software using the scripts table_annovar.pl and annotate_variation.pl, which allows to compare single nucleotide substitutions with the number of specialized databases and to annotate prognostic functional significance of the revealed alterations using six in silico software programs (SIFT, PolyPhen-2, LRT, Mutation Assessor, MutationTaster, phyloP, and GERP++) from dbNSFP v.3.0а. Additionally, we used CLINVAR and CADD (Combined Annotation Dependent Depletion) softwares. Exome sequencing procedure and bioinformatics were performed as previously described by Gilyazova et al. [16].

### 2.5 Network analysis

The network of mutated genes was generated using STRING-DB 9.1 [17]. Minimum required interaction scores were set to “high confidence” (0.700). The Kyoto Encyclopedia of Genes and Genomes (KEGG) pathway enrichment analysis was performed using online KEGG tools (http://www.kegg.jp/).

### 2.6 Statistical analysis

The obtained data was analyzed using R-studio program by calculating average values, standard deviation, and arithmetic mean error. The data are presented as means ± standard deviation. To assess the significance of differences, the Mann–Whitney *U* test was used. The predictive ability of miR‐153 was determined by the receiver operating characteristic (ROC) curves. A logistic regression prediction model was applied to calculate prediction scores for individual samples. Changes were considered reliably significant at p ≤ 0.05.

## 3 Results

### 3.1 MiR-153 expression in PC tissue

**Fig. 1:**
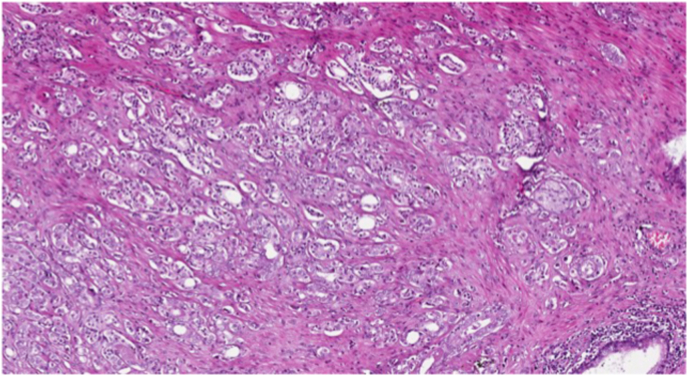
Hematoxylin and Eosin (H&E) stain of prostate cancer (PC) tissue.

**Fig. 2:**
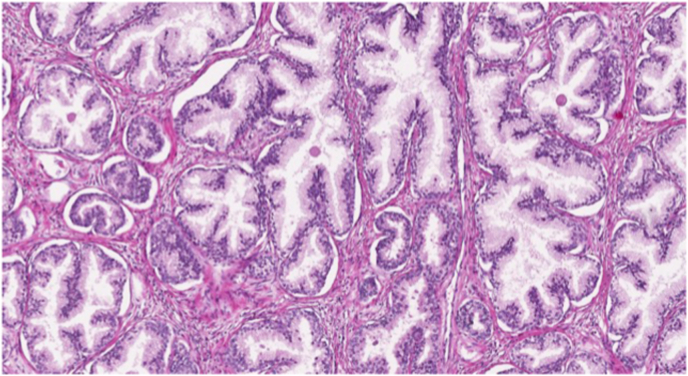
Hematoxylin and Eosin (H&E) stain of normal prostate tissue.

**Fig. 3:**
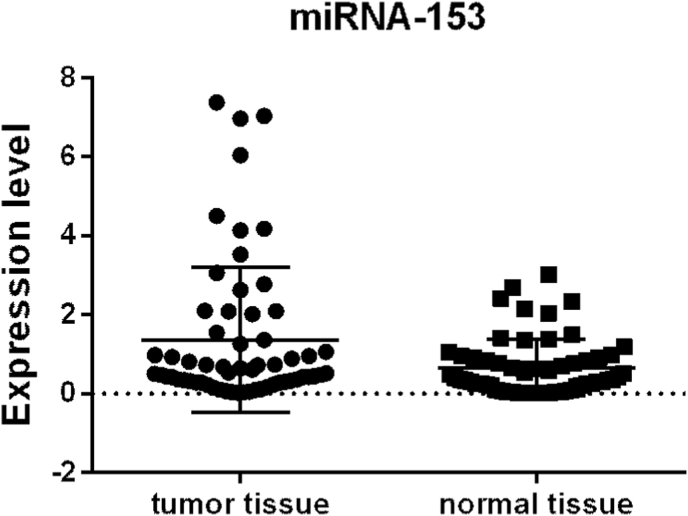
MiR-153 expression level in prostate cancer (PC) tissues vs adjacent normal prostate tissues. A statistically significant increase in miR-153 expression in PC tissue compared to normal tissue was demonstrated. The significance level (p-value) is determined using the Wilcoxon T-test.

The expression level of miR-153 was analyzed in 61 samples of tumor tissue (Fig. 1) and 61 adjacent normal prostate tissue (Fig. 2) using qRT-PCR. MiR-153 expression showed to be increased in tumor tissue (mean ± SEM:1.36 ± 0.23) over normal prostatic tissue (mean ± SEM:0.66 ± 0.09) with p-value = 0.03 (Fig. 3).

### 3.2 MiR-153 expression and clinicopathological factors

**Fig. 4:**
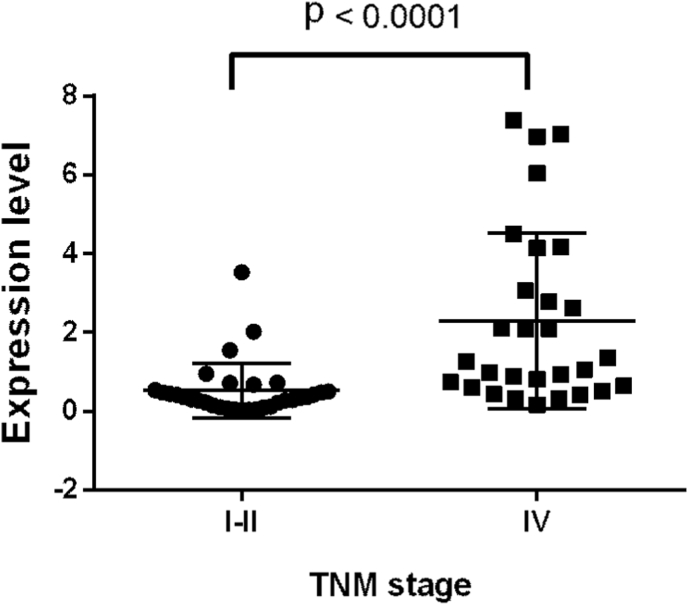
Expression levels of miR-153 in different tumor, node, metastasis (TNM) staging system of prostate cancer (PC). It was demonstrated that miR-153 is upregulated in advanced PC compared to localized disease. Significance level (p-value) was determined using the Mann-Whitney *U* test.

Patients were divided into subgroups with high (n = 31) expression and low (n = 30) expression of miR-153. The median miR-153 expression value defined the threshold value. The analysis of correlation between miR-153 expression and clinicopathological factors (Table 1) revealed a significant correlation with the stage of the tumor process according to TNM classification (p = 0.001). Expression levels of miR-153 in different TNM stages of PC is presented (Fig. 4). It was shown that miR-153 expression significantly higher (mean ± SEM: 2.29 ± 0.41) in metastatic PC tumors compared to non-metastatic tumors (mean ± SEM: 0.53 ± 0.12) with p < 0.0001.

**Table 1:** Association between miR-153 expression and clinical and pathological characteristics of patients with prostate cancer.

Variables	Cases (n)	MiR-153 expression		P-value
		High (n = 31)	Low (n = 30)	
Age, years				
<55	15	9	6	0.818
≥55	46	24	22	
Lymph node metastasis				
Yes	29	22	7	0.180
No	32	18	14	
Bone metastases				
Yes	27	18	9	0.203
No	34	16	18	
Gleason score				
<8	23	12	11	0.710
≥8	38	23	15	
TNM stage				
I + II	32	8	24	0.001
IV	29	23	6	

TNM, tumor, node, metastasis.

### 3.3 ROC curve of miR-153

**Fig. 5:**
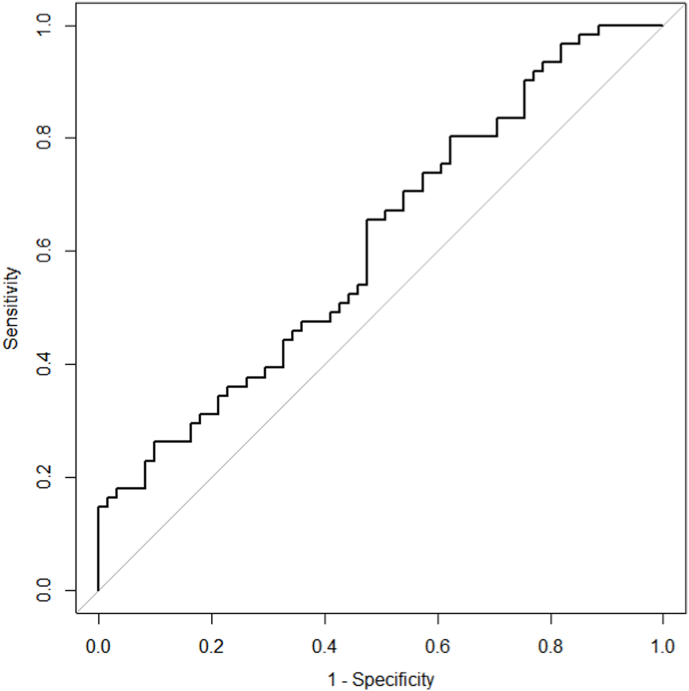
Receiver operating characteristic (ROC) curve analysis of miR-153 to differentiate between prostate cancer (PC) and normal prostate tissue.

In addition, the receiver-operating characteristic (ROC) curve was drawn to further calculate the area under the curve (AUC) and authenticate the diagnostic ability of miR-153. Results revealed that miR-153 expression presented low diagnostic ability for tumor and normal prostate tissue discrimination. As shown at ROC curve of the miR-153 presented in Fig. 5, the AUC was 0.61 (95% CI 0.52–0.71; sensitivity = 0.66, specificity = 0.53). Furthermore, logistic regression analysis revealed that the miR-153 was independent predictor for PC (odds ratios = 1.57, 95% CI 1.13–2.36; p = 0.014).

**Fig. 6:**
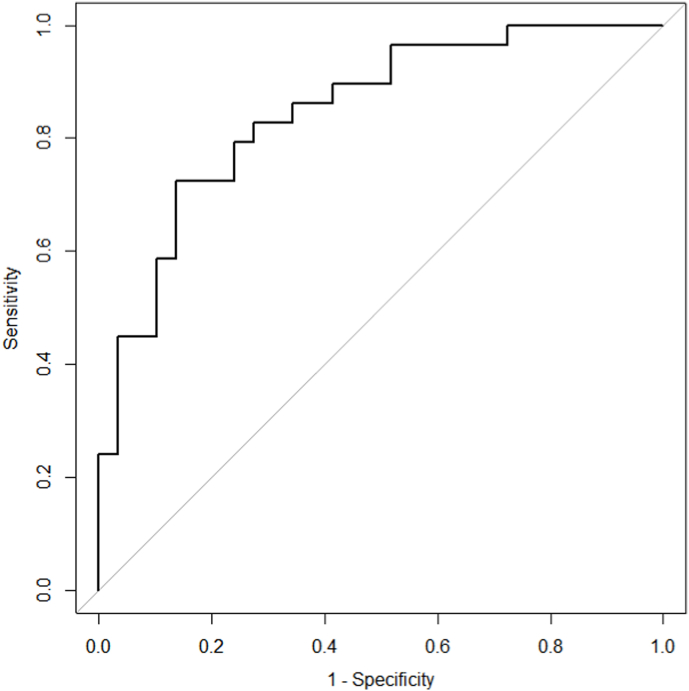
Receiver operating characteristic (ROC) curve analysis of miR-153 to differentiate between metastatic prostate cancer (PC) and non-metastatic PC.

Further the ROC curve analysis was used to evaluate the predictive ability of miR-153 for metastasis development. As shown at Fig. 6 the AUC was 0.85 (95%CI 0.75–0.95; sensitivity = 0.72, specificity = 0.86) that let suggest miR-153 as potential prognostic marker. According to logistic regression model the high expression of miR-153 increased the risk of metastasis development (odds ratios = 3.14, 95% CI 1.62–8.49; p-value = 0.006).

### 3.4 Identification of mutations in miR-153 target genes

In order to identify mutations in miR-153 target genes we performed exome sequencing in all patients with metastatic PC. We identified nonsynonymous somatic mutations in genes collagen type IV alpha 1 (COL4A1), collagen type IV alpha 3 (COL4A3), forkhead box protein O1 (FOXO1), 2-hydroxyacyl-CoA lyase 1 (HACL1), hypoxia-inducible factor 1-alpha (HIF-1A), and nidogen 2 (NID2). No pathogenic mutations were revealed in the coding regions of the phosphatase and tensin homolog deleted on chromosome 10 (PTEN) gene. None of the pathogenic mutations in miR-153 target genes was seen in all patients. This may be due to the fact that shared somatic mutations were not the cause of metastatic PC. The most deleterious germline and somatic mutations are summarized in Table 2.

**Table 2:** Pathogenic mutations identified in target genes for miR-153 using exome sequencing.

ID	Gene	Chr	Pos1	Pos2	Ref	Alt	Hom/Het	SIFT_Score	SIFTpred	MutAssessor	MutTast	PolyPhen2	Phylop
	*ABI2*	chr2	204231743	204231743	A	T	het	0.005	DELETERIOUS	2.27,0.678,M		−0.525	
	*ACTB*	chr7	5567477	5567477	A	G	het	0.001	DELETERIOUS	4.95,0.911,H		2.114	
	*AQR*	chr15	35166090	35166090	A	G	het	0.002	DELETERIOUS	3.54,0.789,H		2.115	
	*AQR*	chr15	35189132	35189132	G	T	het	0.000	DELETERIOUS	3.94,0.823,H		2.743	
	COL4A1*	chr13	110838875	110838875	C	T	het	0.030	DELETERIOUS	2.095,0.663,M	1,1.0,D	0.999,D	2.284
rs115324397	COL4A3*	chr2	228110691	228110691	C	A	het	0.010	DELETERIOUS	2.61,0.708,M	1.000,1.000,D	1.0,D	2.427
	*COPS3*	chr17	17171261	17171261	C	A	het	0.013	DELETERIOUS	2.805,0.725,M		2.572	
	*CSTF1*	chr20	54970669	54970669	C	A	het	0.000	DELETERIOUS	2.12,0.665,M		2.618	
	*EIF3A*	chr10	120802249	120802249	C	A	het	0.008	DELETERIOUS	2.175,0.670,M		2.937	
rs114015346	EPRS*	chr1	220193423	220193423	T	C	het	0.020	DELETERIOUS	3.04,0.745,M	1,1.0,D	0.998,D	1.009
	FOXO1*	chr13	41134822	41134822	C	T	het	0.006	DELETERIOUS	2.8,0.724,M	1,1.0,D	1.0,D	2.763
	*GSPT1*	chr16	11980708	11980708	C	G	het	0.001	DELETERIOUS	2.295,0.681,M		2.584	
	*GTPBP4*	chr10	1038459	1038459	C	A	het	0.002	DELETERIOUS	3.535,0.788,H		2.796	
rs74637339	HACL1*	chr3	15628040	15628040	T	A	het	0.004	DELETERIOUS	2.085,0.662,M	1,1.0,D	0.998,D	2.225
rs61755705	HIF1A*	chr14	62187212	62187212	G	C	het	0.008	DELETERIOUS	2.55,0.703,M	1.000,1.000,D	0.978,D	1.423
	*ITGA1*	chr5	52221259	52221259	C	T	het	0.000	DELETERIOUS	2.095,0.663,M		2.656	
	*ITGAV*	chr2	187505680	187505680	C	A	het	0.003	DELETERIOUS	3.625,0.796,H		2.712	
	*LONP2*	chr16	48311377	48311377	C	A	het	0.004	DELETERIOUS	2.64,0.711,M		2.756	
rs140970953	MCM4*	chr8	48883234	48883234	C	T	het	0.002	DELETERIOUS	4.54,0.875,H	1,1.0,D	1.0,D	2.906
rs61734508	NID2*	chr14	52534758	52534758	C	G	het	0.002	DELETERIOUS	2.325,0.683,M	0.821,0.821,D	1.0,D	1.327
	*ORC1*	chr1	52847394	52847394	G	T	het	0.000	DELETERIOUS	3.795,0.811,H		2.661	
	PBRM1	chr3	52702546	52702546	G	T	het	0.012	DELETERIOUS	3.325,0.770,M		1.449	
	SMAD3	chr15	67477139	67477139	C	A	het	0.000	DELETERIOUS	3.065,0.747,M		2.469	
	YTHDC2	chr5	112891819	112891819	A	T	het	0.000	DELETERIOUS	4.2,0.846,H		0.698	

Note: SIFT: D - disease causing; MutationAssessor: H - high; M − medium; L – low pathogenicity; MutationTaster: D - disease causing. PolyPhen2: D - probably damaging (≥0.957); P- possibly damaging ((0.447 ≤ pp2_hdiv ≤ 0.909); Phylop - the higher is the value, the higher the pathogenicity of the variant; *somatic mutations.

### 3.5 Protein-protein interaction (PPI) network and hub gene analysis

**Fig. 7:**
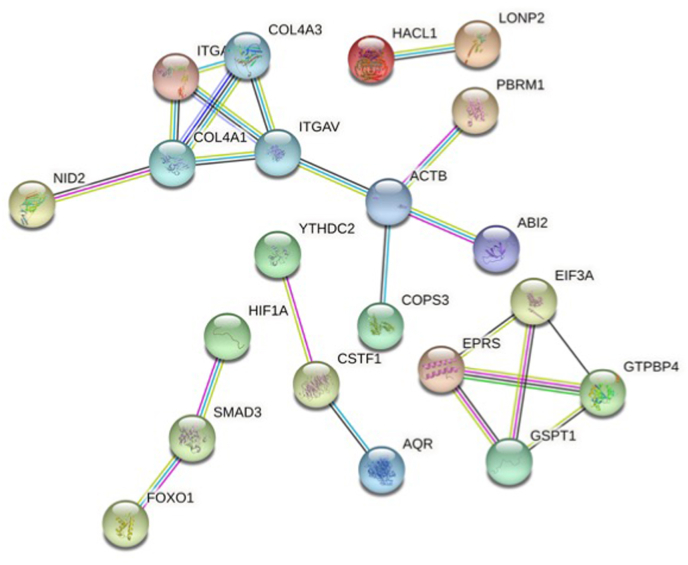
The miR-153-target regulatory interaction network. Protein-protein interaction (PPI) analysis showed that overlapping miR-153 target genes were mainly enriched in collagen type IV trimer, basement membrane, extracellular matrix and cytoplasm.

From 23 proteins, consists of 75 nodes, 23 edges (PPI enrichment value 0.0568, and average local clustering coefficient of 0.253) a PPI network was constructed. Gene Ontology (GO) analysis showed that overlapping miR-153 target genes were mainly enriched in collagen type IV trimer, basement membrane, extracellular matrix and cytoplasm. Regarding molecular function classification, miR-153 target genes were enriched in these functions: transforming growth factor beta binding, ATPase-coupled intramembrane lipid transporter activity, nucleoside monophosphate kinase activity, collagen binding and ATPase activity, coupled to movement of substances. The extracellular matrix–receptor (ECM-receptor) pathway was the most commonly seen enrichment pathway in KEGG (FDR = 0.03) (Supplementary Table S1). STRING analysis of protein-protein interaction network of metastatic PC is presented in Fig. 7.

## 4 Discussion

Patients who have undergone a radical prostatectomy remain at risk of biochemical recurrence after surgery, even though they have increased survival rates [18]. PC prognostic biomarkers are important to facilitate optimization of existing treatment strategies. Recently, it has been shown that miRNAs are intimately related to the development of several different types of cancers and may be useful to determine therapeutic targets for effective treatment strategies in a variety of cancers [19,20]. Xie et al. evidenced that miR-520a inhibits non-small cell lung cancer (NSCLC) progression through suppression of ribonucleoside-diphosphate reductase subunit M2 (RRM2) and Wnt signaling pathways [21]. Dong et al. show that miR-369 expression is reduced in hepatocellular carcinoma (HCC) tissues [22].

MiR-516a-3p expression is suppressed in PC tissue, and loss of miR-516a-3p expression promotes PC progression through ATP binding cassette subfamily C member 5 (ABCC5) targeting [23]. In 2020 Wang et al. presented evidence supporting the fact that miR-1231 expression is decreased in PC tissues and cell lines and that reduced expression of this miRNA had significant association with presence LNM, TNM stage, and clinical stage [24]. This research suggests that miRNAs play an important role in the process of cancer progression.

Previous studies have shown that miR-153 is aberrantly expressed in several common cancers. Zhang et al. performed miRNAs profiling and showed that miR-153 is overexpressed in tissues of advanced colorectal cancer (CC). The miRNAs upregulation was further noted in primary CC compared, unlike normal colorectal epithelium [25]. Further studies showed miR-153 plays a role in promotion of colorectal malignancy progression through invasion and chemotherapy resistance enhancement. Hua et al. showed miR-153 to be activated in HCC cells with correlation between increased expression and poor outcome [26]. In our study we compare expression levels of miR-153 in tumor and normal prostate tissues in patients with metastatic PC and initial stages of the disease and identify specific pathogenic mutations in miR-153 gene targets in PC patients. We showed that miR-153 expression rates are significantly increased in PC tissue, when compared to normal prostate tissue. Moreover, high expression of miR-153 was found significantly associated with TNM stage. Our results suggest that it may act as an oncogene in PC and may be involved in the development of PC.

MiR-153 overexpression is able to stimulate the transcriptional activity of β-catenin, which leads to cycle progression of cells, activation of proliferation and HCC cell colony formation. However, decreased expression of miR-153 is observed in some other cancers. Interestingly, Zhao et al. show that miR-153 expression is suppressed glioma cells compared to normal glial cells [27]. The authors showed that miR-153 suppresses cell invasion by regulating the expression of the snail family transcriptional repressor 1 (SNAI1), which is the target of miR-153. Guo et al. present findings showing that miR-153 is significantly overexpressed in patients with nasopharyngeal carcinoma (NPC), more so this miRNA affects NPC progression through the transforming growth factor-beta 2 (TGF-β2)/Smad2 signaling pathway [28]. Wang et al. showed that miR-153 was overexpressed in BC tissue samples and MDA-MB-231 cells [29]. Our study revealed that miR-153 is in fact overexpressed in PC tissues. Our results are consistent with Wu et al., who identified miR-153 to be overexpressed in PC and showed that miR-153 plays a crucial role in increased proliferation of human PC cells and via a process of miRNA-mediated suppression of PTEN expression in PC cells [8].

So far, there is very limited data on clinical significance of miR-153 and its role in PC. Bi et al. found that high miR-153 expression in PC tissues closely correlated with burdened clinical manifestations [11]. They presented data suggesting that PC patients with high levels of miR-153 expression had a lower five-year survival rate, when compared with patients with low miR-153 expression levels. Importantly, the authors use multivariate Cox regression analysis to show that miR-153 rates of expression are independent factors in predicting 5-year overall outcome in patients with PC. Thus, based on data of the present study and the results of previous publications, we can assume that miR-153 may serve as an available biomarker for PC prognosis.

In our study we built upon existing reports of miR-153 significant by identifying specific somatic mutations in miR-153 target genes (Table 2). One of the mutated genes in metastatic PC patient is COL4A1 gene. COL4A1 is involved in epithelial-mesenchymal transformation (EMT). In previous studies it was found that depending on age of PC patients and Gleason score altered expression of COL4A1 together with 7 other genes may be an EMT marker among PC patients [30]. Mapelli et al. generated a 10-gene predictive classifier which showed that COL4A1, a low-luminal marker, supports the association of attenuated luminal phenotype with metastatic disease [31].

We also found mutations in the FOXO1 gene in metastatic PC patients. The forkhead box O (FOXO) has a common conserved DNA-binding “fork-box” domain and in mammals consists of four members: FOXO1, forkhead box class O 3a (FOXO3a), forkhead box class O 4 (FOXO4), and forkhead box class O 6 (FOXO6). All FOXO factors are involved in a wide range of biological processes, including cell cycle arrest, apoptosis, DNA repair, glucose metabolism, resistance to oxidative stress and longevity [32]. The biological activity of FOXO factors mainly depends on posttranslational modification of phosphorylation, acetylation or ubiquitination, thereby determining their intracellular transport [33]. Dong et al. demonstrated that FOXO1A inhibits androgen receptor (AR)-mediated gene regulation and cell proliferation in PC [34].

Another found mutation in PC cells, minichromosome maintenance complex component 4 (MCM4), belongs to the minichromosomal maintenance (MCM) protein complex which consists of six highly conserved proteins (MCM2-7) collectively interacting to promote DNA replication and DNA unwinding through replicative helicase activity disinhibition [35]. Cancers arising in different anatomic sites are also associated with minichromosome maintenance complex component 2 (MCM2), MCM4, and minichromosome maintenance complex component 6 (MCM6) overexpression, but there is not much information about the role of MCM4 in PC [[35], [36], [37]]. It is known that PSA may mediate MCM4 to promote the initiation and progression of PC and confirmed that PSA knockdown induce the upregulation of MCM4 [38].

Another important tumor microenvironment component is the hypoxia-inducible factor (HIF) pathway. HIF1A was also mutated in metastatic PC patient. There are some articles devoted to analysis of mutations in HIF1A and their role in PC, although ultimately its role in PC remains unknown [39,40].

KEGG analysis showed that a key signaling pathway in metastatic PC is the extracellular matrix (ECM)-receptor interaction signaling. The ECM is a non-cellular component of the stroma of tumor. ECM represent a complex network of macromolecules which undergo extensive reconstruction during tumor progression. Such remodeling of the extracellular matrix during cancer progression causes changes in its density and composition [41]. Damage to the ECM structure leads to reactive growth of tumor cells due to switching of intracellular signaling processes and cell cycle changes [42]. Proliferation increases, normal tissue architectonics is lost, local migration of tumor cells and invasion into surrounding stromal tissue occurs. One of the main factors determining the degree of tumor malignancy is the process of EMT characterized as loss of epithelial phenotype by epithelial cells and acquisition of mesenchymal phenotype associated with the ability to migrate through basal membrane. ECM is accompanied by loss of cell adhesion molecule E-cadherin, cytokeratins, increased N-cadherin, fibronectin, and vimentin [43]. ECM proteins provide biochemical signals to induce EMT. In turn, EMT becomes an inducer of metastasis, triggering various transcription factors. Thus, transformation plays an important role in tumor progression and metastasis, involving various transcription factors (TFS) and signaling processes [43,44].

## 5 Conclusions

Our results show that high miR-153 expression is associated with TNM stage increase in PC patients. Several potential PC related genes and pathways were identified in the study, which will improve our understanding of the molecular mechanisms which support prostate cancer progression and development. A key signaling pathway, the ECM-receptor interaction signal pathway, was identified as possibly involved in the development of PC. Further investigations are needed to perform in-depth analysis on gene ontology, browser tracks, and expression levels of miR-153 targets in metastatic PC patients.

## Compliance with ethical standards

Ethical approval for this study was obtained from Institute of Biochemistry and Genetics Bioethics Committee. The study was carried out in accordance to Helsinki Declaration and local guidelines.

## Funding

This work was supported by the Megagrant from the 10.13039/501100017638Government of Russian Federation № 075-15-2021-595, the 10.13039/501100003443Ministry of Education, and Science of the Russian Federation № 122041400169-2. In the study, DNA samples from the “Collection of Biological Materials of Human Beings” of the IBG UFRC RAS were used, supported by the Program of Bioresource Collections of the 10.13039/501100013176FASO of Russia [Agreement No.007–030164/2]. This work was supported by the Bashkir State Medical University Strategic Academic Leadership Program (PRIORITY-2030).

## Informed consent

All samples investigated in this study were obtained with written informed consents of the participants.

## Availability of data and material

All supporting data and materials are available from the corresponding author upon reasonable request.

## Author's contribution

Irina Gilyazova: conceptualization, writing–original draft, and project administration. Elizaveta Ivanova: writing–review and editing, investigation. Mikhail Sinelnikov: formal analysis, writing–review and editing, methodology. Ilgiz Gareev: resources and data curation. Aferin Beilerli, Ludmila Mikhaleva, and Yanchao Liang: validation and data curation. Valentin Pavlov and Elza Khusnutdinova: validation and visualization. Valentin Pavlov and Elza Khusnutdinova: supervision and funding acquisition. All authors have read and agreed to the published version of the manuscript.

## Declaration of competing interest

The authors declare that they have no known competing financial interests or personal relationships that could have appeared to influence the work reported in this paper.
